# Multiferroics under the tip: probing magnetoelectric coupling at the nanoscale

**DOI:** 10.1093/nsr/nwz056

**Published:** 2019-05-09

**Authors:** Yunya Liu, Jan Seidel, Jiangyu Li

**Affiliations:** 1 Key Laboratory of Low Dimensional Materials and Application Technology of Ministry of Education, School of Materials Science and Engineering, Xiangtan University, China; 2 School of Materials Science and Engineering, University of New South Wales, Australia; 3 ARC Centre of Excellence in Future Low-Energy Electronics Technologies, University of New South Wales, Australia; 4 Shenzhen Key Laboratory of Nanobiomechanics, Shenzhen Institutes of Advanced Technology, Chinese Academy of Sciences, China; 5 Department of Mechanical Engineering, University of Washington, USA

Multiferroic materials, with the coexistence of electric and magnetic orderings and thus exhibition of magnetoelectric coupling, offer an intriguing playground for controlling and understanding the structure of condensed matter interacting with external stimuli, and promise exciting opportunities for technological applications. While originally envisioned in the 1950s, the field of multiferroics took off in the last two decades, wherein the advances in theory and computation, materials processing and film deposition, as well as structure and property characterization have fueled the appreciation of new understanding, discovery of new phenomena, development of new materials, and emergence of new devices. Of particular interest to us is the emergence of scanning probe microscopy (SPM) techniques, which turns out to be a powerful yet readily accessible tool to probe and manipulate multiferroics at the nanoscale [1,2]. In this perspective, we highlight the opportunities and challenges, and provide an outlook of multiferroics under a SPM tip.

**Figure 1.**
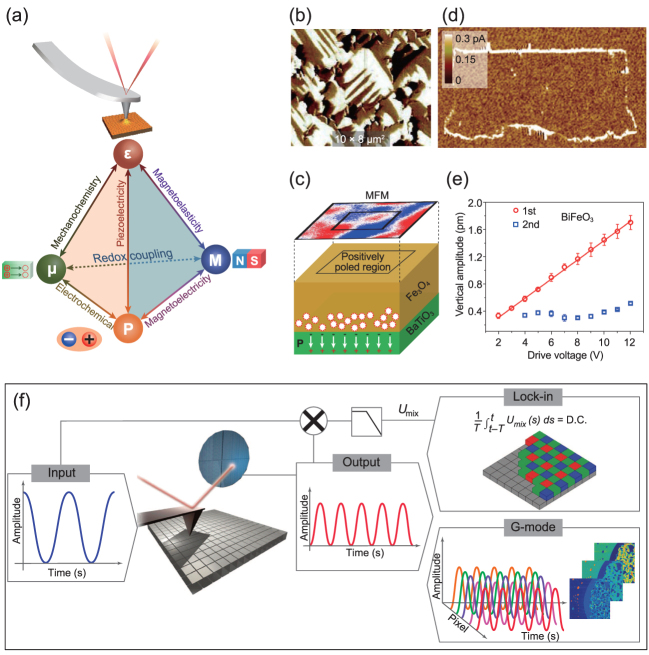
Scanning probe microscopy (SPM) of multiferroic materials with magnetoelectric coupling. (a) The schematic probing of multi-field coupling among mechanical, electrical, magnetic, and chemical fields. (b) Piezoresponse force microscopy image of a single-phase BiFeO_3_ film. Reprinted with permission from Ref. [3]. (c) Magnetic force microscopy image of a BaTiO_3_–Fe_3_O_4_ heterostructure. Reprinted with permission from Ref. [6]. (d) Conductive atomic force microscopy image of domain walls in BiFeO_3_. Reprinted with permission from Ref. [11]. (e) Comparison of the first and second harmonic piezoresponses of BiFeO_3_. Reprinted with permission from Ref. [16]. (f) The schematics of G-mode SPM for big data. Reprinted with permission from Ref. [19].

The electric and magnetic orders in a multiferroic are intimately coupled to strain, and recently it has also been realized that they can be manipulated and controlled via electrochemical means as well, such as ionic migration and redox reaction. The scanning probe as schematically shown in Fig. 1a provides a set of convenient techniques to probe a variety of order parameters and their coupling in multiferroics. For example, by exciting piezoelectric strain arising from spontaneous polarization via a charged probe, the ferroelectric ordering can be studied by piezoresponse force microscopy (PFM) [1,2], which turns out to be instrumental in probing single-phase multiferroics such as bismuth ferrite [3] shown in Fig. 1b, and it is also possible to probe the electrochemical process via Vegard strain [4]. When combined with computational studies such as the phase field method [5], which simulates ferroelectric ordering at the same length scale as probed by PFM, deep insight can be gathered on the configuration and evolution of ferroelectric domains.

In parallel, investigations of magnetic ordering at the nanoscale can be accomplished using magnetic force microscopy (MFM) via magnetostatic interaction between the sample and a magnetized tip. This is mostly carried out in multiferroic heterostructures consisting of ferroelectric and ferromagnetic constituents [6], as shown in Fig. 1c, since single-phase multiferroics do not usually possess strong magnetism at room temperature. Low-temperature MFM has been carried out to study magnetic ordering [7], which could be useful for studying single-phase multiferroics. Originally developed by Xie *et al*. [8], the evolution of PFM response under external magnetic field has been widely used to probe magnetoelectric coupling at the nanoscale, and the configuration has recently been extended to probe the local Hall effect [9]. Magnetoelectric force microscopy (MeFM) has also been proposed by combining MFM with *in situ* modulation of high electric fields [10].

Combined with other SPM techniques, such as conductive atomic force microscopy (c-AFM), it is possible to study the influence of electric and magnetic order on various other properties, e.g. local conductivity on the nanoscale. The discovery of conductive domain walls in multiferroics [11], as shown in Fig. 1d, has spawned a new research field, and led to investigations of prototype nanoelectronics elements based on domain walls [12]. The controlled formation of domain wall junctions and defined geometries have led to the exploration of vortex–antivortex pair structures [13], which combined with electric readout through conductive domain walls or vortex cores could enable new memory and data storage concepts. Multiferroics in general offer new approaches for research on topological structures on the nanoscale, allowing one to exploit order parameter coupling. Some recent examples include skyrmions in multiferroic lacunar spinels [14] and 2D multiferroic materials [15].

While substantial progress has been made on multiferroics under the tip in the last 20 years, significant challenges remain. Such local probing is prone to the influence of artifacts and cross-talk, and a variety of electromechanical coupling effects exist in complex media [2], including the piezoelectric effect, Vegard strain, electrochemical dipoles, and electrostriction. Comparing the first and second harmonic piezoresponses turns out to be very effective in distinguishing true linear piezoelectric strain from other mechanisms that are often non-linear in nature [16], as demonstrated in Fig. 1e. It has also been shown that apparent magnetic contrast can arise from electrostatic interaction [17], and thus it is important to probe the sample using oppositely magnetized tips. Caution also needs to be exercised when comparing piezoresponse under different magnetic fields, especially for quantitative analysis, as the response is usually magnified near resonances, and a correction based on dual amplitude resonance tracking (DART) via a simple harmonic oscillator (SHO) model [18] is not very reliable. This points to a new direction for big and deep data [18,19], as well as machine learning and artificial intelligence [20], which can be effective in reducing noise, discerning artifacts, identifying dominant microscopic mechanisms, and enabling more reliable quantitative analysis. A new line of thinking known as G-mode is to excite the probe via arbitrary signals instead of sinusoidal waves, as schematically shown in Fig. 1f, and acquire full time-domain data instead of utilizing lock-ins at particular frequencies [19]. Alternatively, data acquisition can be achieved in a more targeted manner, such as sequential excitation (SE) [18], which provides more data points than DART for higher-fidelity SHO analysis. The advances in the data aspect of SPM, in combination with realistic modeling and simulation [21], could make accurate quantitative measurement possible at the nanoscale.

It is also critical to continue pushing the spatial resolution for polarization and magnetization imaging. This is particularly important for probing in multiferroics that possibly host magnetic skyrmions as well as polar vortex and bubble structures. Currently these intriguing textures are mostly imaged at the atomic scale via high-resolution transmission electron microscopy (TEM), within which an electric probe can be utilized to manipulate the polar order. It is also possible to probe magnetic ordering using a tip equipped with a nitrogen vacancy (NV) center, as demonstrated in real-space imaging of non-collinear antiferromagnetic order in BiFeO_3_ [22], and this could enable mapping of magnetic skyrmions with higher resolution. In a multiferroic with magnetoelectric coupling, it is conceptually possible to image polar vortices or bubbles as well. Realizing such capabilities will enable exciting studies of local interactions as well as dynamics in such intriguing systems.
